# Puerperal Insanity

**Published:** 1903-02-07

**Authors:** 


### PUERPERAL INSANITY.

At the last meeting of the Obstetrical Society, an interesting discussion on puerperal insanity took place, ranging over a large amount of ground, and touching incidentally upon many topics. A matter of considerable importance, which was dealt with by several speakers, was as to the nature of the early symptoms. Dr. Robert Jones described the early symptoms of threatened insanity as loss of sleep and headache. Dr. Blandford said that beyond question the earliest symptoms of approaching mental trouble in these cases was loss of sleep. He held that this should be most closely watched, and that every precaution should be taken that the patient should not be disturbed by noises or talking in the room, by the child being brought to nurse, or by the visits of friends. Dr. Herman thought that the main duty of the general practitioner and of the obstetric physician in regard to puerperal insanity was to prevent it, treatment being mainly in the hands of the alienist. To prevent puerperal insanity the great things were to see that the patient got food and sleep, and in regard to the latter point he thought that if sleep were absent the best hypnotic was alcohol. There were objections to the use of alcohol of which in the present day no one was in danger of losing sight. Indeed, every hypnotic if taken too much did harm, but the harm done by alcohol if taken too freely was far less and far slower in coming than that done by chloral, bromide, morphine, sulphonal, or any other hypnotic if taken habitually for long periods. Dr. Mercier took exception to the statement that headache was a common prodroma of a puerperal insanity. The only reliable indications were, he thought, sleeplessness and loss of appetite. Dr. Champneys fully endorsed all that had been said about sleeplessness as the striking symptom in threatening insanity, about the necessity of procuring sleep, and about the pre-eminent value of alcohol as a sedative in such cases. Dr. Walter Griffiths regarded the refusal of food as of equal importance with sleeplessness as a premonitory symptom, and he was sure that in general practice the absolute necessity of forced feeding, even by the nasal tube, was not properly recognised as the essential means of saving life. In this view the President (Dr. Peter Horrocks) agreed, saying that refusal of food was a far graver symptom and pointed much more certainly to insanity than did sleeplessness. This, and headache too, occurred not infrequently in patients who never developed insanity, whereas refusal of food, and by that was meant not mere anorexia, was practically only associated with insanity. In regard to treatment the importance of removal to a special institution was insisted upon by several speakers. Dr. Seymour Tuke laid down three points in favour of asylum *versus* home treatment; first, that it was hard upon the patient to be under control where she had hitherto been in command; secondly, the possibility in a well ordered asylum of instantly changing the attendants if necessity arose through the patient taking a dislike to any of them; and thirdly, the fact that a home where restraint of any kind had been applied was likely to have less pleasant associations and memories after recovery. He also, like other speakers, urged the necessity of good feeding, and of the early resort to artificial feeding if called for; but in regard to alcohol, while allowing its great utility, he thought that the danger of forming a habit must not be lost sight of.

